# Development of Total Lymphoid Irradiation (TLI)-Dedicated Shielding and Image-Guided System and Dose Evaluation Using 3D-Printed Rat Phantom

**DOI:** 10.3389/fvets.2022.832272

**Published:** 2022-05-18

**Authors:** Dong Hyeok Choi, So Hyun Ahn, Kwangwoo Park, Sang Hyun Choi, Jin Sung Kim

**Affiliations:** ^1^Department of Medicine, Yonsei University College of Medicine, Seoul, South Korea; ^2^Department of Radiation Oncology, Yonsei Cancer Center, Yonsei University College of Medicine, Seoul, South Korea; ^3^Department of Radiation Oncology, Yongin Severance Hospital, Yonsei University College of Medicine, Yongin, South Korea; ^4^Research Team of Radiological Physics and Engineering, Korea Institute of Radiological and Medical Sciences, Seoul, South Korea

**Keywords:** total lymphoid irradiation, position-controlled shielding system, *in vivo* dosimetry, image-guided radiotherapy, 3D-printed phantom, rat

## Abstract

**Purpose:**

The purpose of this study is to propose a technique for delivering accurate doses in an image-guided system by developing an experimental setup optimized for total lymphoid irradiation (TLI) in rat lung transplantation.

**Materials and Methods:**

In this study, a position-controlled shielding system was developed, and the dose was quantitatively evaluated using a 3D rat phantom and Gafchromic EBT3 film. In addition, we made our own image-guided system that allows the position of the rat and the shielding system to be confirmed during TLI.

**Results:**

As a result of using the position-controlled shielding system, it was found that the doses to the head and lungs were reduced by 93.1 and 87.4%, respectively, of the prescribed doses. In addition, it was shown that the position of the shielding system can be easily confirmed by using the image guidance system.

**Conclusion:**

A shielding apparatus that can control dose delivery according to the size of the rat can optimize the dose for TLI in rat lung transplantation.

## Introduction

Allogeneic transplantation should be preceded by animal experiments, and vascularized mouse lung transplantation has been only recently developed, unlike other solid organs (1). Total lymphoid irradiation (TLI) is essential to increase the safety and efficacy of allografts.

We can expect that TLI reduces the incidence of bronchiolitis obliterans, which is a common adverse effect induced by inappropriate immunosuppression or changes in the immune system after lung transplantation (2). It was successfully used to prevent renal and cardiac allograft rejection following transplantation (3). The immunosuppressive properties of TLI have been documented specifically for the reduction of the T-cell population (4, 5).

Efforts have been made to reduce unnecessary doses to the lungs and brain in total body irradiation, which is performed to suppress autoimmunity before allogeneic stem cell transplant in human acute leukemia patients prior to TLI in animals (6).

The most commonly used animals for lung transplantation experiments are mice and rats. Accurate anatomical and physiological modeling is an important basis for clinical research. Xie et al. reported a literature study on rat or mouse models developed in 1994 (7). Animal models are used for preclinical studies, and since the early 1990s, more than 120 animal models have been developed. According to the authors, the voxel model, quadratic equation-based model, and hybrid model have been used.

In addition, there have been many studies on the accuracy improvement of animal irradiation experiments. Bax et al. made a 3-dimensional image-guided robotic needle positioning system for small animal interventions to reduce the targeting error to <200 μm (8). The authors noted that using this device, image-guided radiation therapy (IGRT) could be used for preclinical biomedical applications. Kim et al. developed a mouse mold with a 3D printer to improve the accuracy and reproducibility to irradiate the mouse brain with CyberKnife (9). They were able to track using fiducial markers in the 3D mold when irradiated with CyberKnife and showed positional accuracy of 1.41 ± 0.73 mm.

A number of TLI studies have been performed on mice and rats (10, 11). Hoogenhout et al. evaluated the accuracy of dose delivery and evaluated shielding when performing TLI on a rat or mouse (10). They developed a rat phantom with Masonite and irradiated the beam with two anterior ports using a 250-kVp X-ray machine. Maximum dose measurements at the center of the field and dose distributions within the Masonite (density: 1.08 g/cm^3^) rat phantom were performed with TLD and file exposure. The rat phantom produced by the authors did not reflect the 3D anatomy of the rat and was made in a flat form for film insertion.

Halperin et al. performed TLI on a cardiac allograft model of rats and observed heart graft survival times (GST) using a cobalt-60 machine (11). They found that GST was increased from 6.9 ± 0.3 days up to 25.5 ± 1.1 days when TLI was performed. However, GST tended to decrease when 20 Gy or more was irradiated, because of irradiation-induced pulmonary toxicity and host death. They used lead alloy block to shield the head, tail, most of the lungs, and half the liver. In addition, tissue-equivalent gelatin rat phantom and thermoluminescent dosemeter (TLD) were used for dose evaluation.

Studies related to the combination of IGRT in radiotherapy for small animals have been continuously conducted. Recently, IGRT technology for the treatment of tumors in small animals is evolving from the conventional approach that involves delivering a uniform beam with a simple shielding approach (12). In addition, many radiation therapy research centers are developing new research platforms that can investigate radiation responses from tumors and normal tissues in small animal models. The Small Animal Radiation Research Platform (SARRP) has developed a portable system for optimally planned radiation through detailed irradiation with a beam size of about 0.5 mm and an onboard conical beam CT (CBCT) guide (13). The microIGRT system was developed to study the radiological effects of isometric radiation distribution in small animals, and the system, X-Rad 225Cx, consists of a flat amorphous silicon detector mounted on 225 kVp x-ray tubes and rotational C-arm gantry and can image fluoroscopy x-rays and cone-beam CT (14). Using a preclinical radiation research platform, a study was conducted that proposed guidelines for technical issues to be considered for radiation therapy research (15). In this study, using the Xrad-320 irradiator model without a gantry, it is not possible to take a cone beam image.

The IGRT technology and application to small animals are evolving from the existing approach [Small animal image-guided radiotherapy]. In the IGRT study related to small animals, a quantitative analysis was conducted on the effects of respiratory motion in relation to the delivered dose distribution (16, 17). In addition, studies have been conducted considering the IGRT approach to understand the response of tumors to radiation (18), studies using small animal IGRT as an MRI-based radiation therapy scheme for mouse tumors (19). However, there are still institutions that conduct small animal experiments using an irradiator without an imager. When these institutions continue to irradiate rats for several weeks, it is impossible to adjust the field size of radiation to the rats that have grown and changed in size. If the rats can be observed and the position of the shielding apparatus can be changed, the accuracy of the experiment will be significantly improved. Therefore, we have developed 1) a rat CT image-based 3D-printed rat phantom for *in vivo* dose verification and 2) developed a position-controlled shielding system that can be flexibly adjusted when the size of the rat grows during the TLI period. In addition, a self-developed image-guided system was used to improve the positioning accuracy of the rat and shielding apparatus.

## Materials and Methods

### Experimental Conditions of Xrad-320 Irradiator

**Figure 1:**
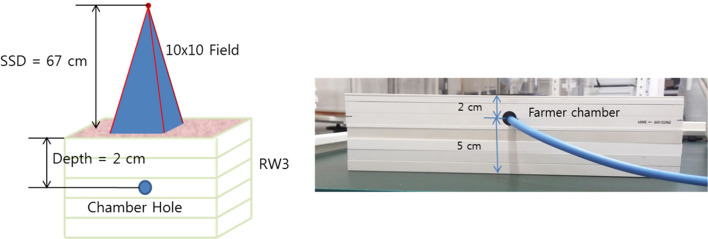
Experimental set-up for absolute dose measurement under the following reference conditions: field size 10 x 10 cm, 320 kVp, 12.5 mA, irradiation time 30 s, SSD 67 cm, backscatter materials 5 cm, depth 2 cm, and 2 mm Aluminum filter.

The TLI and absolute calibration were performed by Xrad-320 (Precision X-Ray, USA) with 320-kVp tube voltage and 12.5-mA tube current. For accurate dose delivery, the absolute dose calibration is essential, which followed the instruction of task group report 61 (TG-61) protocol (20): The absolute dose was measured at the 67 cm source-to-surface distance (SSD) and 10 X 10 cm field size using the Farmer type chamber (Type 30013, PTW, Germany) with the water equivalent solid slab phantoms (Figure 1). In our institution, the ionization chamber is calibrated at a standard national standard institution every 6 months. In the rat phantom experiment, the SSD was set to 50 cm to cover the rat's whole body above the image-guided device, which will be described he sections II-4. In addition, a 2 mm Al filter was used for calibration and dose measurement. To determine prescription depth, the percent depth dose (PDD) was measured with the water equivalent solid slab phantoms. The voltage, current, and exposure time to acquire the image are the same as the set-up criteria for delivering the prescribed dose. The dose delivered per second (Gy/s) was calculated by dividing the dose irradiated for 1 min by 60 considering the ramp-up time. The ramp-up time (δt) was obtained using the mathematical method (Equation 1) mentioned in the TG-61 equation (20). We took three measurements and averaged them to get the ramp-up time. Table 1 shows the data for calculating the δt of the irradiation equipment, and finally, δt was calculated to be 0.95 s. Accordingly, 0.95 s was applied as a fixed value for all measurements.


(1) $$\begin{array}{r} \delta_{t}\;=\;\frac{M_{2}\Delta t_{1}\;-\;M_{1}\Delta t_{2}}{M_{1}\;-\;M_{2}} \end{array}$$

**Table 1:** The table shows the dt results for the exposure time and chamber readings as the ramp-up time (δt) calculation data for the irradiation equipment.

**Number of measurements**	**t1**	**t2**	**M1**	**M2**	**δt**
1	30	60	12.35	24.27	1.08
2	30	60	12.31	24.27	0.88
3	30	60	12.35	24.34	0.90
Mean	30	60	12.34	24.29	0.95

*A total of three measurements were taken and averaged*.

Where *M*_1_ and *M*_2_ are the chamber readings for exposure time Δ*t*_1_ and Δ*t*_2_, respectively.

### 3D-Printed Rat Phantom

**Figure 2:**
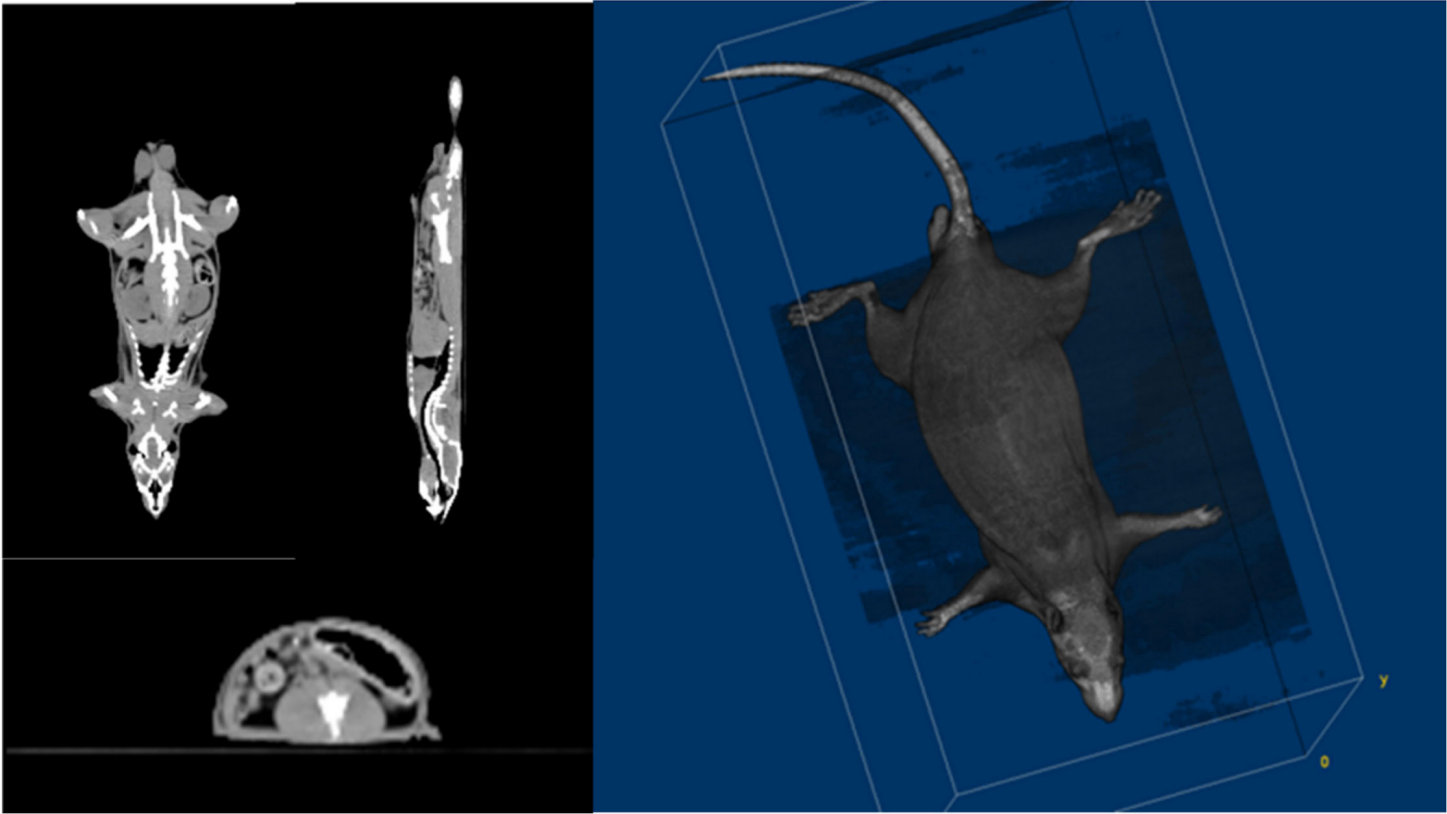
CT images of rats, including axial, coronal, sagittal, and its 3D rendering images.

**Figure 3:**
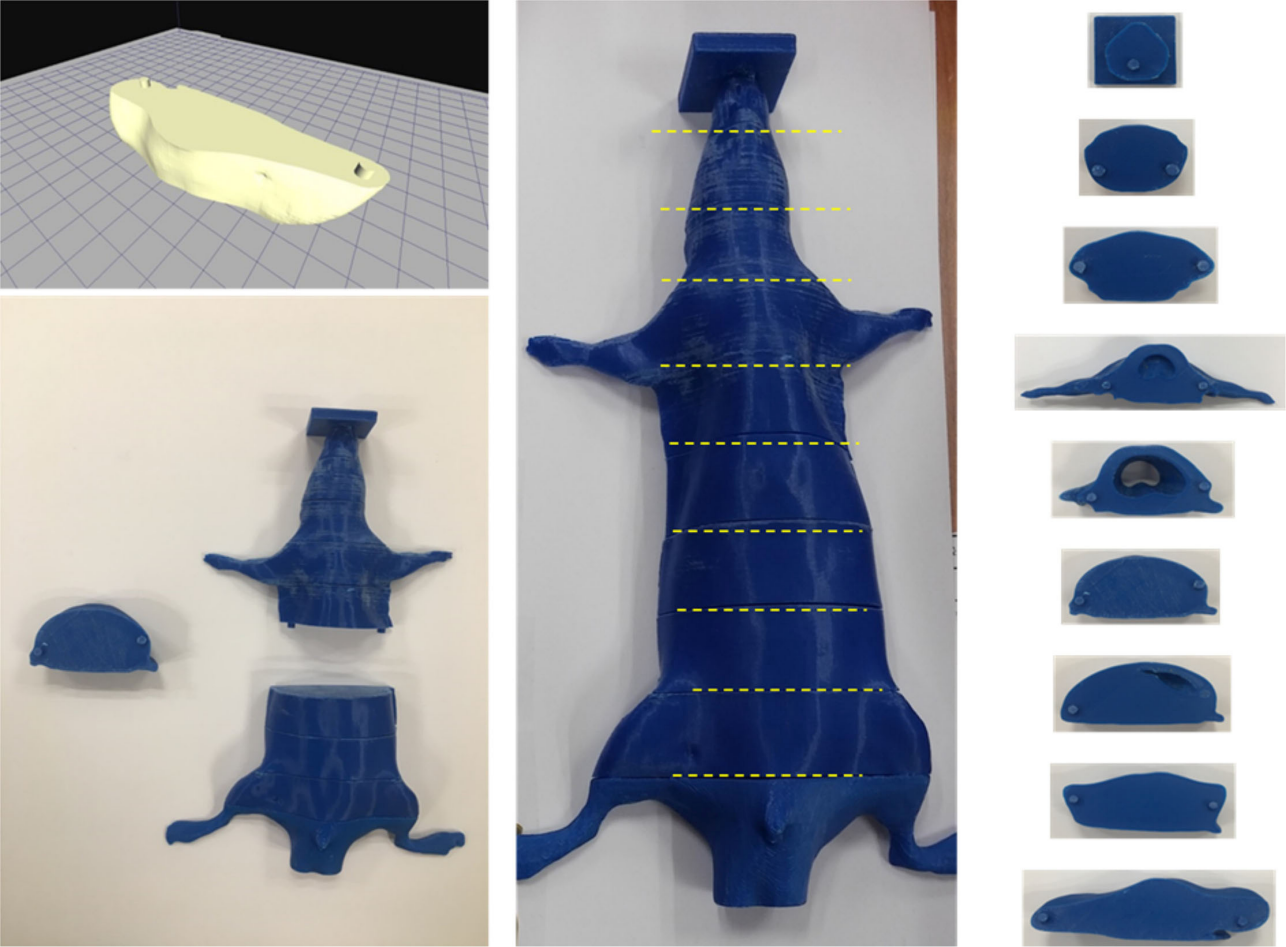
A slice combined rat phantom which can be inserted film into each slice using a 3D printer.

Small animal CT is suitable for use with rats because of its high resolution. However, the rat's limbs should be folded and placed on the torso side and placed in a semi-cylindrical fixed frame. This posture is actually not the same as that of the rat when performing TLI. Therefore, the CT images were obtained with conventional CT (Siemens SOMATOM Definition 128-slice CT scanner). Figure 2 shows the axial, coronal, sagittal, and 3D rendering CT images of the rat used to make a 3D-printed rat phantom with the filament of polylactic acid (PLA, ρ: 1.25 g/cm^3^). The phantom was made based on the CT images of a 10-week-old rat. Verifying the shielding effect, the internal dose of rats was evaluated. For this purpose, a rat phantom was prepared in the form of a slice so that the EBT3 film could be inserted (Figure 3).

### Gafchromic EBT3 Film

The Gafchromic EBT3 film was scanned on an Epson Expression 10000XL scanner 48 h after irradiation. To maintain constant reproducibility, 5 pre-scans were performed before the first scan, and the film was fixed using a compression plate to prevent movement or lifting of the film. A calibration curve for the response of the EBT3 film was created by fitting the scan results of the EBT3 film obtained using various doses. After scanning as a red-green-blue (RGB) image, to reduce the scan error within the scanner field in all measurements, the scan results were obtained by changing the position of the film, and the average value was used. For each scanned image, 600 dpi RGB images were acquired for analysis. Using ImageJ software, a 4 cm^2^ region of interest (ROI) was set at the center of the film to obtain the RGB average value of the image intensities.

### Position-Controlled Shielding System

**Figure 4:**
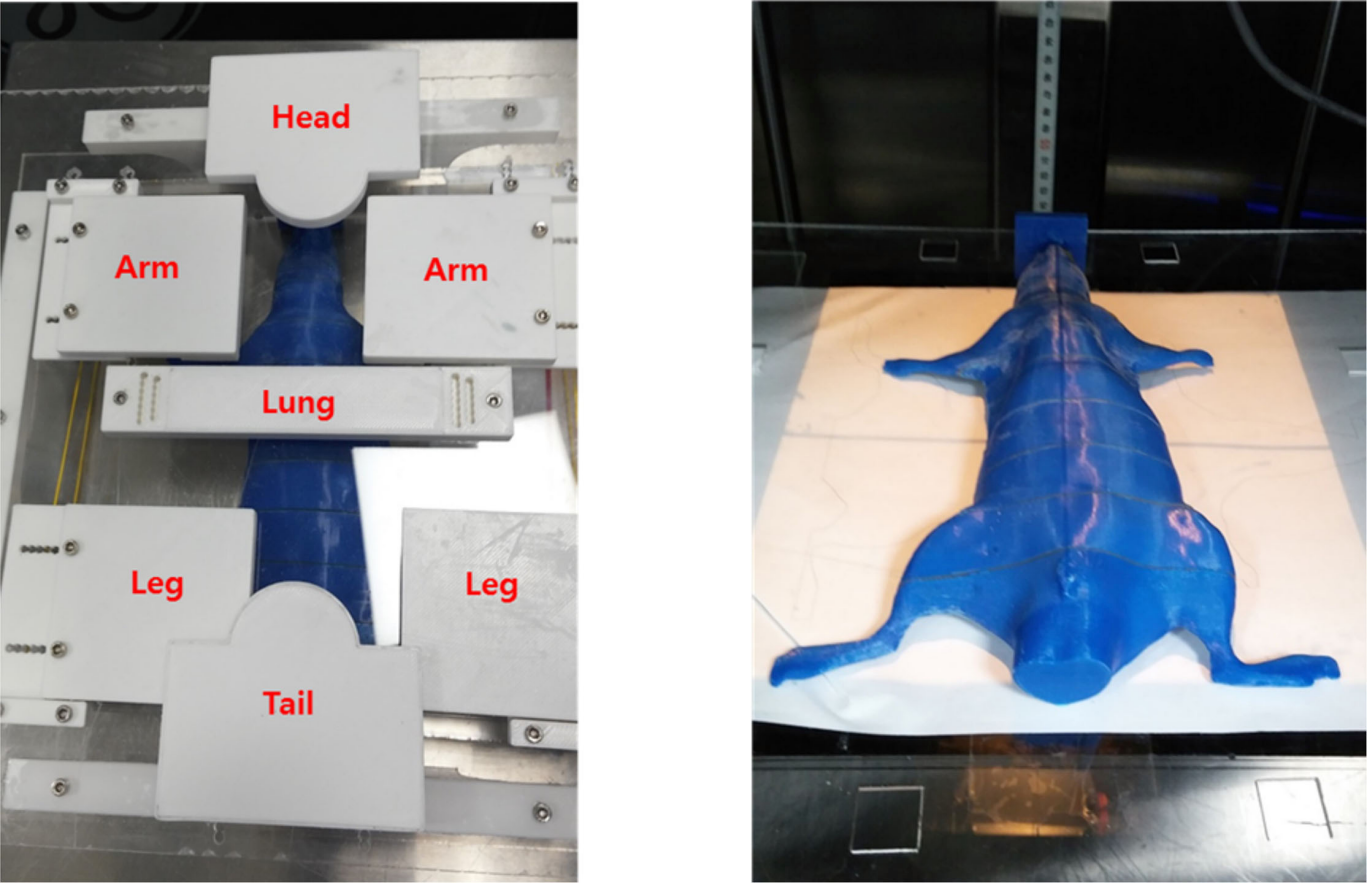
The shielding apparatus is fabricated in a flexible manner to adjust the position of shielding materials considering the size change due to the growth of rats during total lymphoid irradiation (TLI) implementation.

A shielding apparatus is required for areas where irradiation is unnecessary. In addition, TLI lasts for 4 weeks, during which the rat grows. Therefore, the shielding system created at the beginning of the first TLI may need to be resized in the later part of the TLI. Thus, we needed to fabricate a position-controlled shielding system that could change the size to match the growth of the rat. The shielding apparatus was fabricated into five parts that can be assembled: head, arm, lung, leg, and tail (Figure 4). Each part was mounted on an acrylic structure so that it could be flexibly moved according to the size or position of the rat. The material used for the shielding is Cerrobend (50% Bismuth, 26.7% Lead, 13.3% Tin, 10% Cadmium), and the thickness is 2 mm. After melting the Cerrobend, it was poured into the white polylactic acid (PLA) cover as shown in Figure 4.

### Image-Guided System

**Figure 5:**
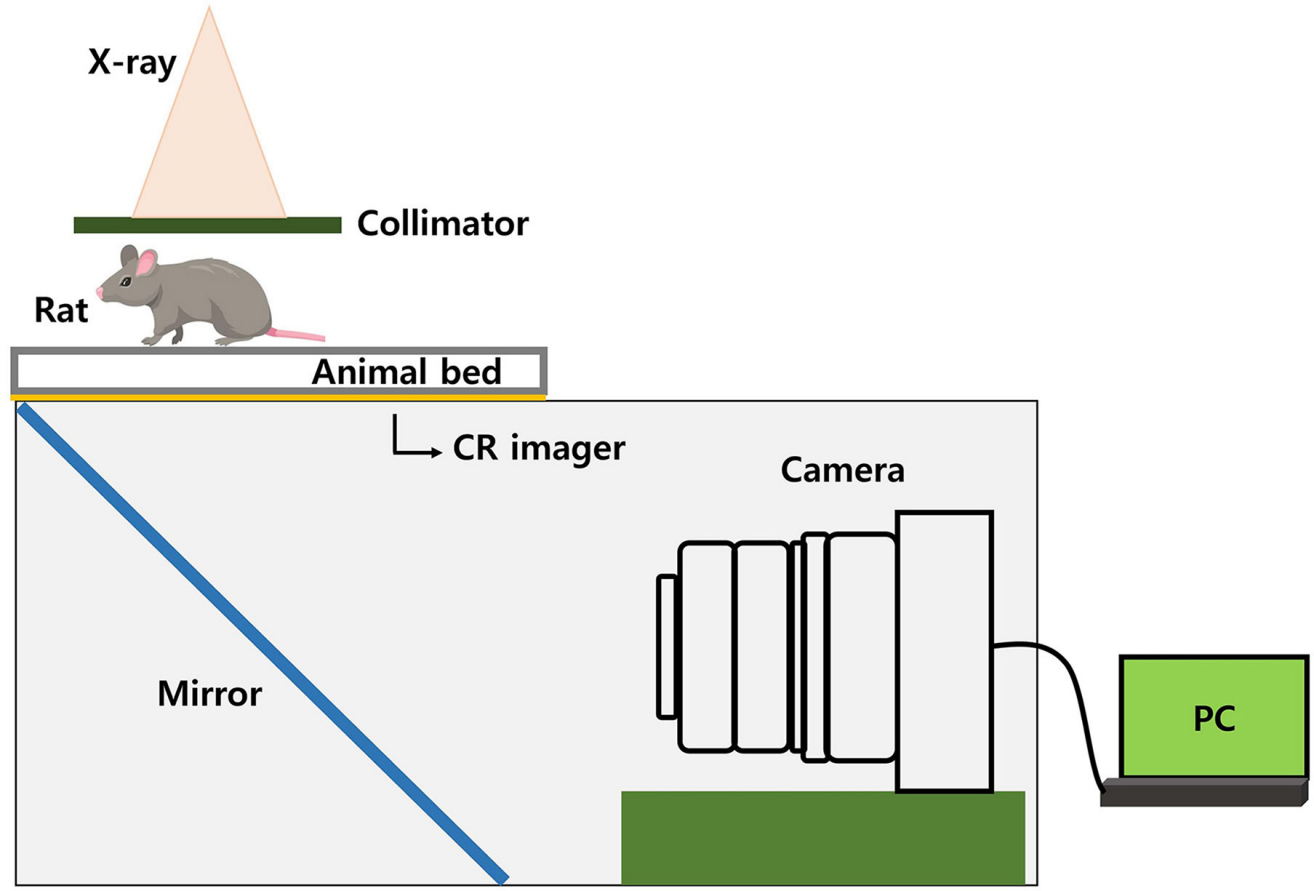
Image guidance system made using CCD camera.

In the case of TLI using a shielding system, the reproducibility of the position between the rat and the shielding apparatus is important. Therefore, we used an image-guided system, which was made using a charge-coupled device (CCD) camera. By irradiating a small amount of radiation (0.122 Gy) for imaging, the shielding apparatus and the position of the rat can be verified. We made our own image-guided system, not a commercial one. The conceptual diagram of the image-guided system is shown in Figure 5. We used a CR imager (Codak MIN-R 2000) that makes visible image and reflect *via* a mirror to the camera. What is reflected in the mirror is an x-ray image that has passed through the rat and entered the CR imager. So, our camera system avoids direct beam irradiation using a mirror. Besides, an imaging panel (including rat-experimental-panel) was installed over a 2D linear motion stage which can be moved by a stepper motor and controlled by a computer.

In the case of TLI using a shielding system, the reproducibility of the position between the rat and the shielding apparatus is important. Therefore, we used an image-guided system, which was made using a charge-coupled device (CCD) camera. By irradiating a small amount of radiation (0.122 Gy) for imaging, the shielding apparatus and the position of the rat can be verified. Our camera system avoids direct beam irradiation using a mirror.

## Results

**Figure 6:**
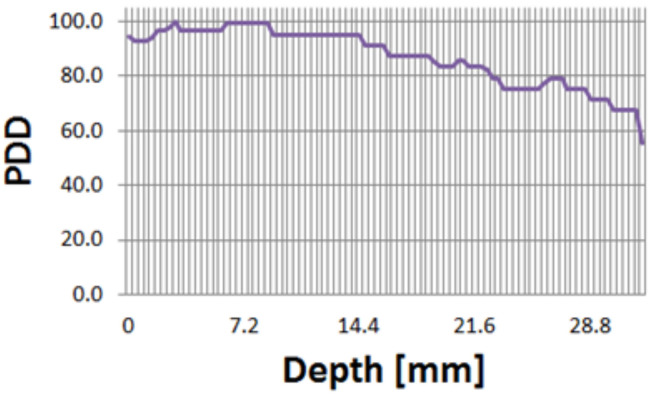
Percent depth dose measurements with the solid water phantom.

As a result of absolute dose measurement, a dose rate of a Gy/s was measured. Also, as a result of PDD measurement, it was confirmed that 80% of the maximum dose was delivered to a depth of 2 cm in the solid water phantom (Figure 6). Considering that the Anterior-Posterior 1 port is used in TLI, 2-cm depth was selected as the prescription point rather than the midpoint.

**Figure 7:**
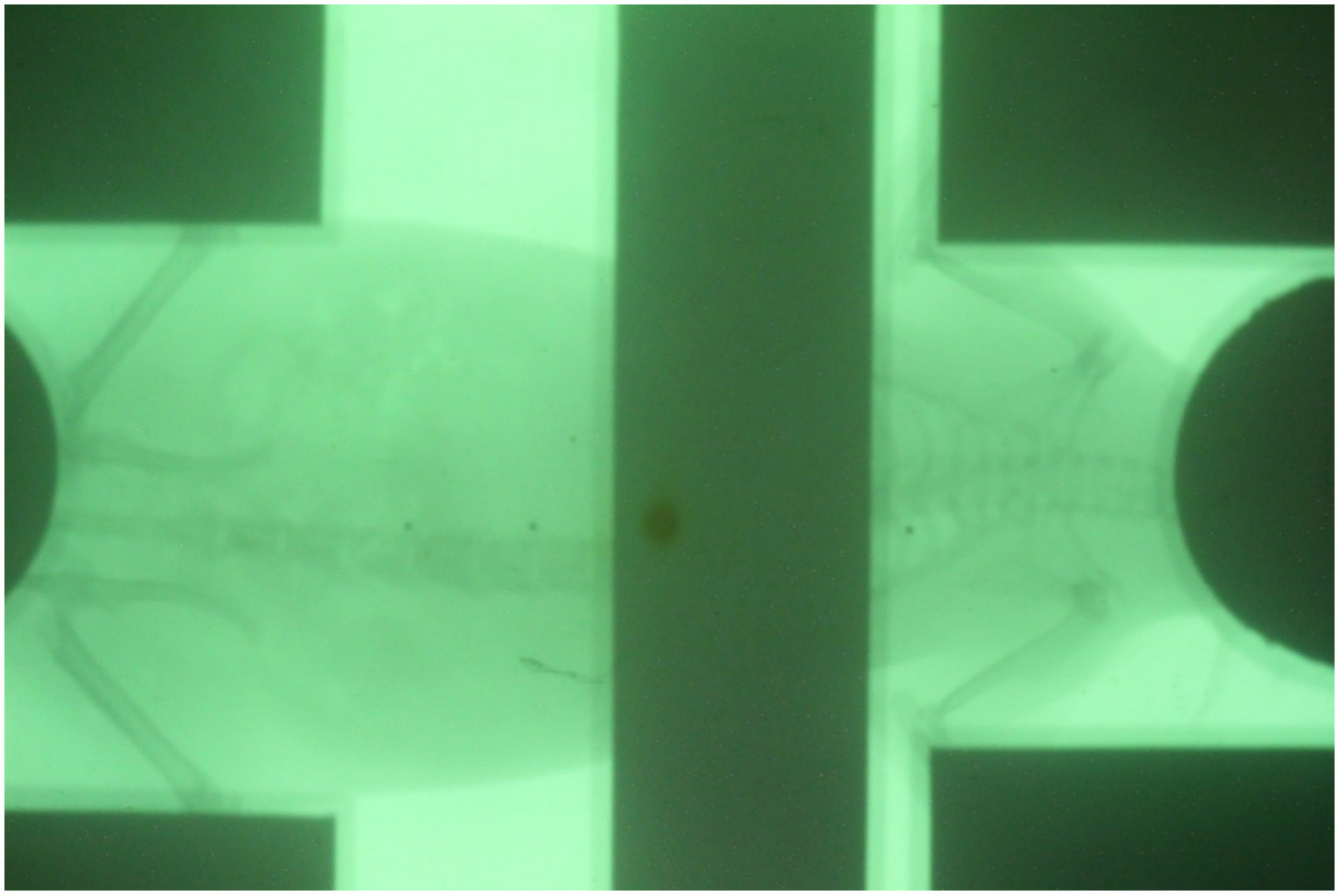
Verification of the position of shielding apparatus and rat positioned by image-guided system.

**Figure 8:**
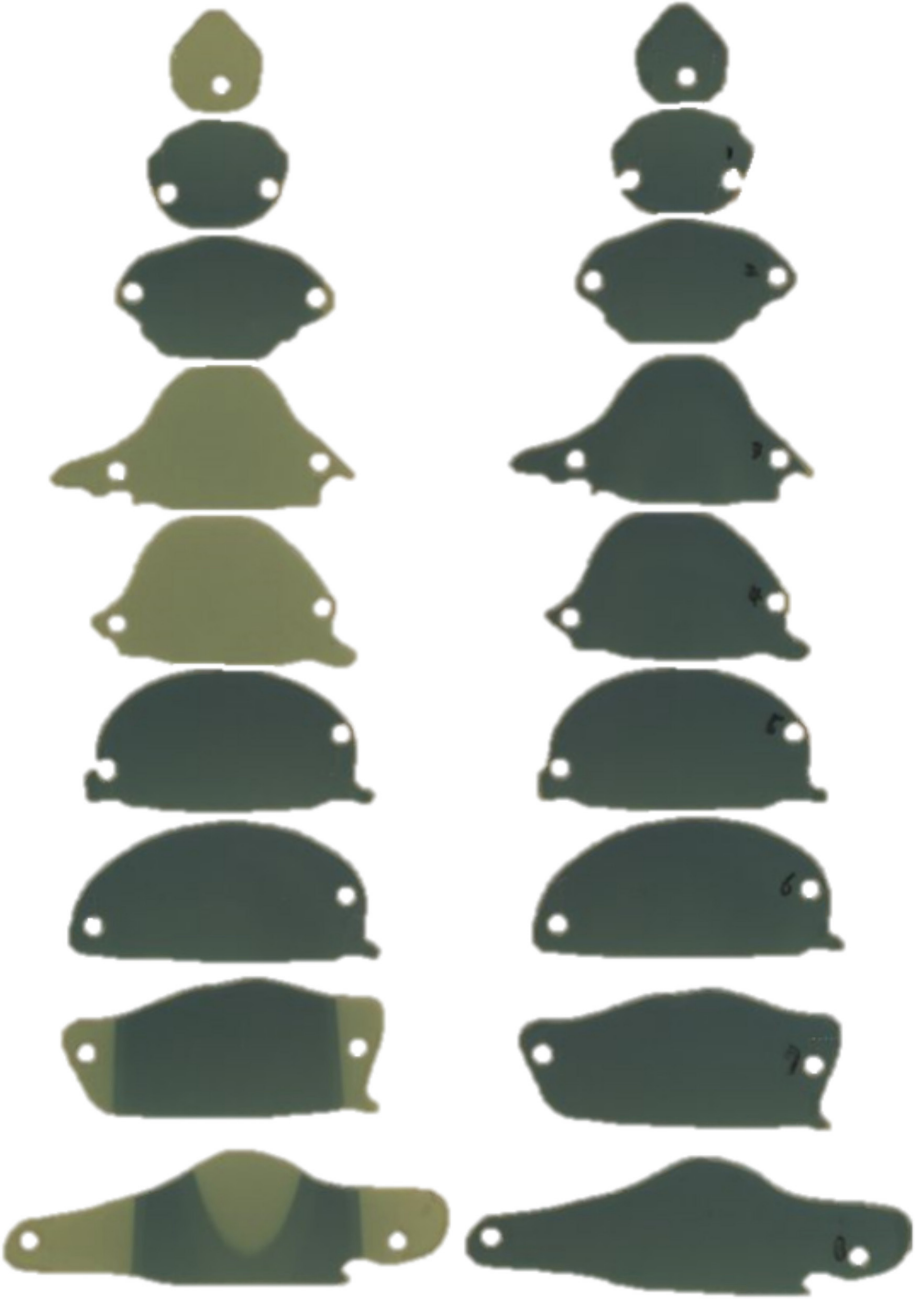
The film was inserted into each slice of the rat phantom to measure dose (**left**) when using and (**right**) when not using a shielding apparatus.

After the shielding apparatus and rat phantom were set up, the image-guided system was used to verify that the position of the shielding apparatus is correct, as shown in Figure 7. The shielding apparatus is connected by screws, and the size can be adjusted by changing the screw connection position according to the size of the rat. When using a shielding system (Figure 8), it was found that the head decreased by 92.7% (0.22 Gy) compared to the prescribed dose (3 Gy) and decreased to 86.7% (0.4 Gy) compared to the prescribed dose in the lung (Table 2). The use of the shield apparatus reduced the upper and lower lungs by 74.4 (2.23 Gy) and 87.3% (2.62 Gy) compared to the prescribed dose, respectively. The lower lung dose attenuation is greater. It was verified that the dose of lung and head can be effectively reduced in the case of rat TLI using shielding apparatus.

**Table 2:** The difference between the measured dose and the prescription dose in the film was inserted into each piece of the rat phantom according to the use of the shielding apparatus.

**Site**		**With shielding apparatus**		**Without shielding apparatus**	
	**Shielded**	**Measured dose (Gy)**	**% difference** **(from prescription dose)**	**Measured dose (Gy)**	**% difference** **(from prescription dose)**
Head	Y	0.22	92.7	2.52	16.2
Neck		2.22	25.9	2.43	19.0
Shoulder		2.15	28.4	2.76	8.1
Lung (upper)	Y	0.46	84.6	2.69	10.2
Lung (lower)	Y	0.40	86.7	3.02	−0.6
Abdomen (upper)		2.44	18.6	2.88	3.9
Abdomen (lower)		2.60	13.4	2.89	3.6
Hip (upper)		2.48	17.2	2.93	2.2
Hip (lower)	Y	0.51	82.9	2.64	12.0

*TLI was performed by Xrad-320 (Precision X-Ray, USA) with 320-kVp tube voltage and 12.5-mA tube current at 2 cm depth*.

## Discussion

Since the system proposed in this study uses a model without a gantry, it cannot take cone-beam images. However, we have independently implemented an alternative camera system capable of performing IGRT, which potentially provides a suitable solution with a lower cost standard irradiator and greater throughput.

Our institution is using the TG-61 protocol for dose calibration of the X-rad320 irradiator. We performed the experiment to compare the results of dose calibration with the TRS-398 protocol (21). Table 3 shows the factors applied when converting the dose using two protocols. For the TRS-298 protocol, a PTW 0.125-cm^3^ Semiflex chamber with k_Q_ factor of medium energy X-ray was used. The absolute dose rates were measured as 0.022 Gy/s and 0.023 Gy/s using TG-61 and TRS-398 protocols, respectively. Therefore, using the TRS-398 protocol, the dose was converted to 5% higher than when using the TG-61 protocol. It can be verified that the dose difference between the TG-61 and the TRS-398 protocol is about 5%, but it remains difficult to determine which of the two protocols represents a value close to the actual dose. In this study, we continued to use the existing TG-61 protocol.

**Table 3:** Parameters applied to TG-61 and TRS-398 protocols.

**Factor**	**TG 61**	**Factor**	**TRS 398**
Equation	$D_{w,z=2\;cm}=MN_{K}P_{Q,cham}P_{sheath}{\left[{\left(\frac{\mu_{en}}{\rho }\right)}_{air}^{w}\right]}_{water}$	Equation	*D*_*w,Q*_ = *M*_*Q*_*N*_*D,w*,_*Q*__0__*k*_*Q*,_*Q*__0__
Ion chamber (model)	Farmer (TM30013)	Ion chamber (model)	Semiflex (TN31010)
Depth	2 cm	Depth	2 cm
SSD	67 cm	SSD	67 cm
P_sheath_	1.0 (no water proofing sleeve)	K_Q,Q0_	1.015 (for 0.2 mm Cu HVL)
N_k_	0.04882 Gy/nC	N_D,W_	0.3015 Gy/nC
P_O,Chm_	1.021 (for 0.2 mm Cu HVL, for 2 cm depth)		
${\left[{\left(\frac{\mu_{en}}{\rho }\right)}_{air}^{w}\right]}_{water}$	1.076 (for 0.2 mm Cu HVL, for 2 cm depth)		
Phantom	RW3	Phantom	RW3

As shown in Figure 4, the parts that are actually shielded are the head, lung (upper, lower), and the hip (lower). When the shielding apparatus was used, the average measured dose was reduced by 2.32 ± 0.2 Gy (77.2 ± 6.7% of prescription dose) compared to when it was not used. In addition, when the shielding apparatus was used, it was confirmed that the average decrease of 0.4 ± 0.15 Gy (13.3 ± 5% of prescription dose) was observed in other areas that were not shielded. When no shielding apparatus is used, it can be seen that the dose distribution gradually decreases with respect to the front of the beam exiting. When the shielding apparatus was used, a maximum dose of 2.62 Gy (87.3% of prescription dose) was reduced in the lung located in front of the beam direction but decreased by 2.14 Gy (68.0% of prescription dose) in the hip (lower) farther away from the beam direction.

In general, when measuring dose using film or other detectors, it is necessary to repeat the measurement twice or more to prevent biased results due to unexpected statistical error, but in this experiment, we performed film measurement only once. This shows that there is no problem in evaluating the usefulness of a shielding system that can be positioned according to the growth of rats, but it acts as a limiting point in terms of value as a scientific paper.

The *in vivo* dosimetry of realistic dose distributions in the body is always an important issue in the field of radiation therapy. Research on the method of analyzing the 3-dimensional dose distribution measured by gel dosimetry (22, 23) or transit dose (24, 25) during the treatment has been actively studied. However, lower dosimetric accuracy has been applied for small animal experiments than that required for human patients. In this study, the 3D-printed phantom was developed based on the CT images of the rat used for TLI experiments, and the dose distribution in the rat phantom was evaluated. In this study, 3D printing was performed with PLA material. In this study, a very precise dose distribution could not be created because an irradiator was used rather than a piece of treatment equipment equipped with MLC. Accordingly, an experiment was conducted for the purpose of measuring the dose to a prescribed point at a specific point in the body without considering high atomic number substances, such as bone. The phantom geometry is similar to that of the actual rat and is closer to a more realistic one than the existing animal studies. Therefore, it is considered that the value of the present study is increased in terms of a more accurate evaluation of dose distribution for small animal experiments. However, the density of the PLA substance differs from that of the rat, and the substance of the lung is also implemented as an empty (air-filled) space for the convenience of production since there is no bone material, such as the spine. The dose measured using the developed phantom may be different from the actual dose of the rat, although the developed phantom is elaborated based on CT images of the actual rat.

Using a 320 kVp, 12.5 mA, and 2 mm Al filter on the X-rad 320 irradiator device, the nominal HVL is 1 mm Cu (26). In this study, dosimetry was performed with reference to the value suggested by TG-61, and parameters for the X-ray beam were set based on the nominal HVL. In TG-61, ${\left[{\left(\frac{\mu_{en}}{\rho }\right)}_{air}^{w}\right]}_{water}$ for HVL 1 mm Cu is presented as 1.076, and the values of P_O,Chm_ are presented as 1.023. Accordingly, 9.9% of the dose was corrected during measurement in this study.

The TLI for a patient may be divided into two fields or a combination of two different energies because a large field is required and the depth to which the radiation should be irradiated varies from region to region (3). However, the TLI for the rat reduces the need to combine multiple fields, because the depth of the rat is not deep, and the field size is sufficient to include the whole body of the rat. Hoogenhout et al. used two anterior portals for TLI of the rat (10). One portal encompassed lymph nodes above the diaphragm, including the thymus, and the other encompassed lymph nodes below the diaphragm, including the spleen. Halperin et al. also set up the rat in the anterior direction and used one portal (12). We set up the rat in the most natural prone position and delivered the radiation using the posterior 1 port.

The image-guided technique is a key part of modern radiation therapy. The Xrad320 irradiator provides a field light that allows you to view the radiation field during setup. Using the field light to set up is an intuitive and convenient method when uniformly irradiating a cell plate or the whole body of the mouse. In recent years, however, small animal experiments have been conducted to improve the outcome of experiments, such as irradiating or shielding only certain areas. In this sense, more sophisticated experiments will be possible if reconstructed images, such as 3-dimensional cone-beam CT, used in patients are used. However, this is expensive and is not available in small animal testing setups in most hospitals. We aimed to improve the accuracy of small animal experiments by using an image-guided system that can observe fluoroscopy in real-time, although it is a two-dimensional image.

To set up the shielding apparatus and the image-guide, the total experimental time of the day is approximately 30 min more. However, continuous beam irradiation is virtually impossible due to the nature of the equipment, which requires cooling time. Therefore, there is no change in the number of rats that can be tested because there is no actual increase time because the image-guide is prepared and the shielding apparatus is adjusted during the cooling time. The biggest influence on the number of rats that can be irradiated in 1 h is the cooling time, and approximately 10 rats can be tested per hour.

**Figure 9:**
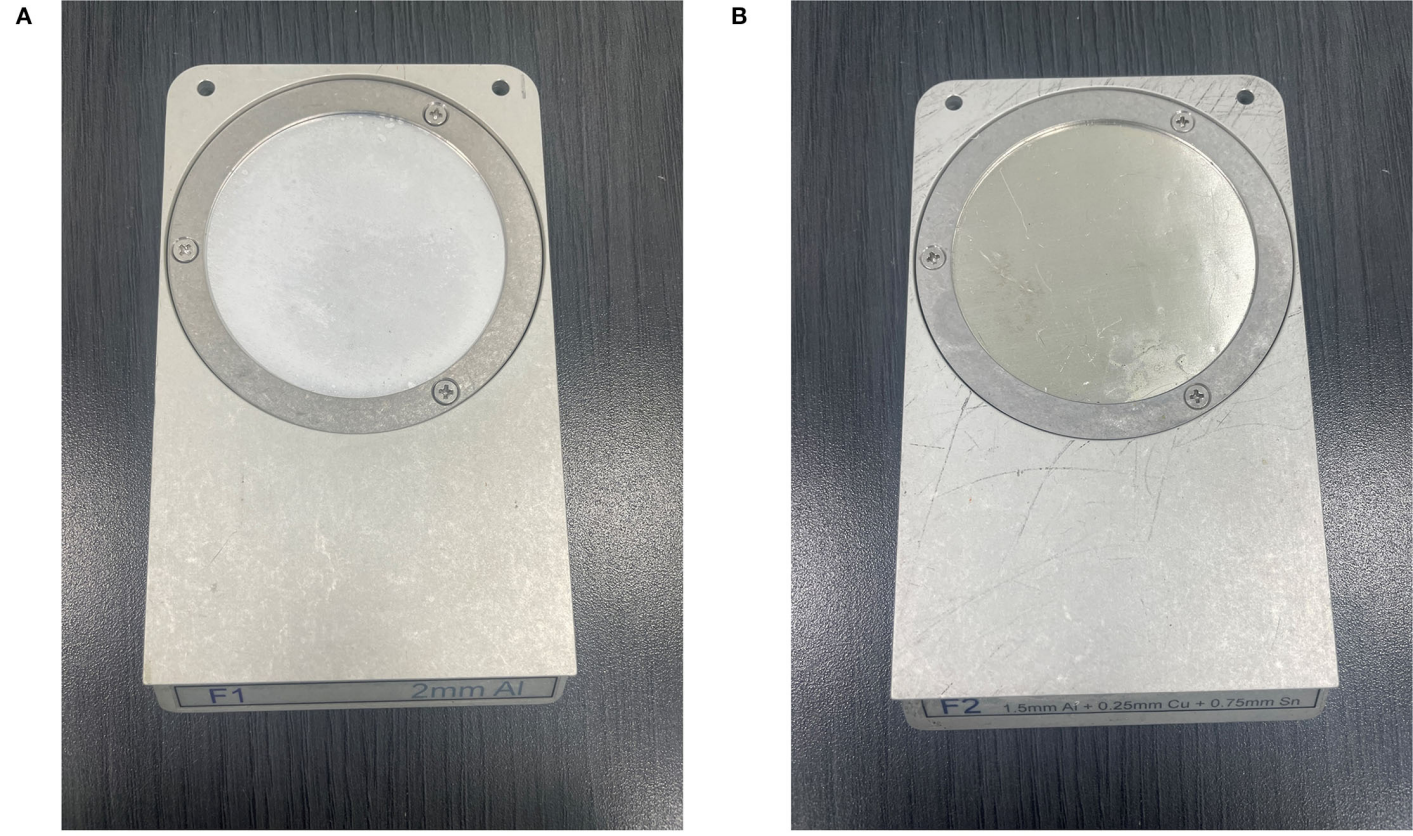
Filtration filters used in X-rad 320 irradiator **(A)** F1 filter (2 mm Al), **(B)** F2 filter (1.5 mm Al + 0.25 mm Cu + 0.75 mm Sn).

**Figure 10:**
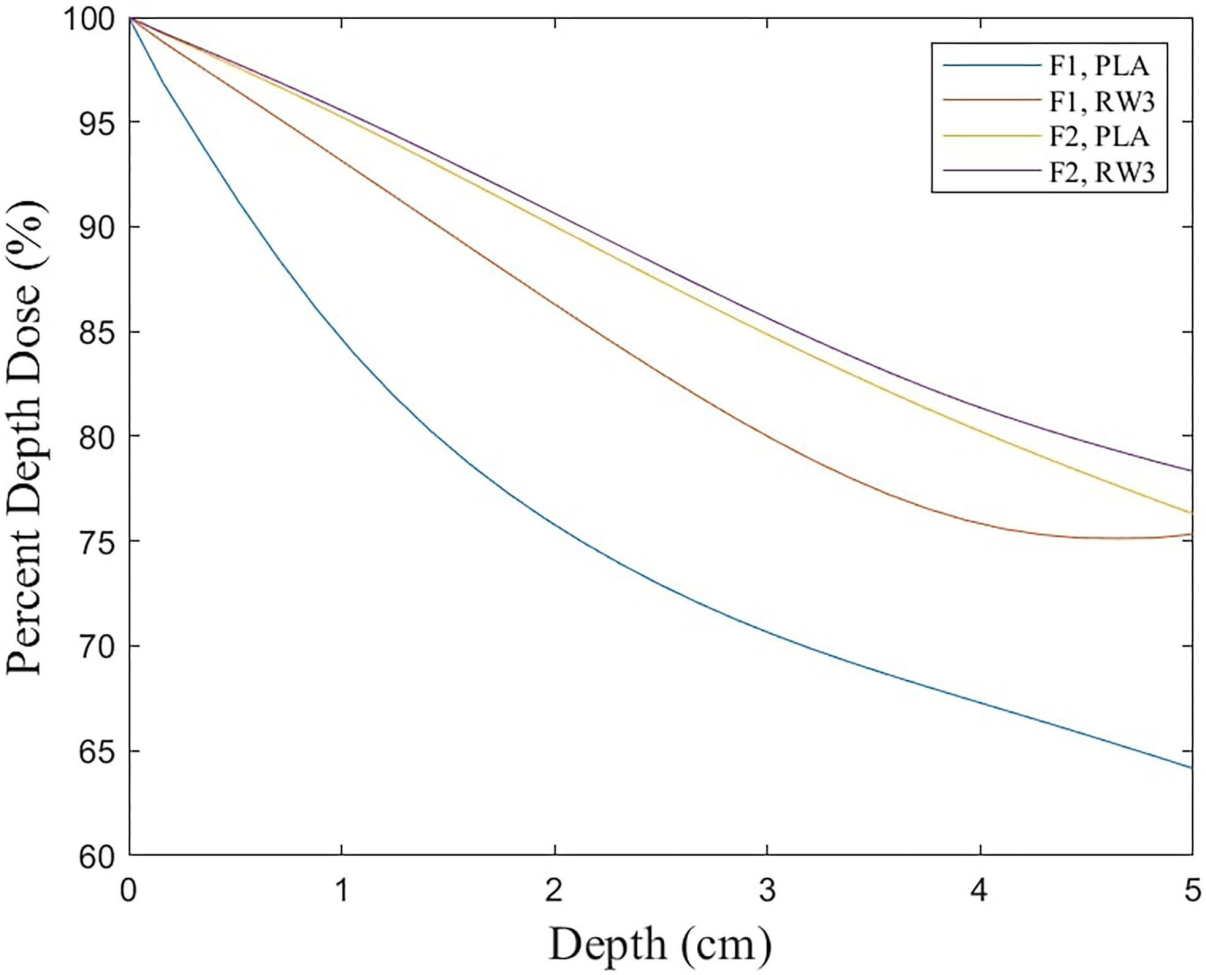
Results of percent depth dose up to 5 cm depth.

The Xrad320 irradiator we used in the experiment has an F1 filter (2 mm Al) and an F2 filter (1.5 mm Al + 0.25 mm Cu + 0.75 mm Sn) (Figure 9). In the initial research meeting, our medical physics team explained the beam sorting and hardening for the two filters at the surgery and xenograft research meeting, and the surgical team selected 2 mm Al for the low-dose issue. It was incorrectly stated that 1 mm of Al was used in the text, so it was corrected to 2 mm of Al. To confirm the effect of filtration according to the filter, the percent depth dose and the uniformity of the dose distribution according to filtration were quantitatively evaluated using F1 and F2 filters in PLA and RW3 phantom. PDD and dose distribution were performed by Xrad-320 with 320-kVp tube voltage and 12.5-mA tube current at 2 cm depth. PDD for PLA and RW3 phantom was measured for two filters up to 5 cm depth of the phantom using EBT3 film, respectively (Figure 10). When the F1 filter was used, PDD showed a steeper curve than when the F2 filter was used. As you mentioned, this additional experiment confirmed that the skin dose can be relatively reduced in the F2 filter compared to the 2 mm Al filter. Based on these results, we will refer to choosing between the two filters according to the prescription depth in future small animal experiments.

For dose distribution, EBT3 film scan results were obtained for a total of 9 ROIs of 1 × 1 cm using EBT3 film 5 × 5 cm. As a result of using the F1 filter, there was a difference in dose distribution of about 3% in PLA phantom and about 1% in RW3 phantom, and as a result of using the F2 filter, there was a difference in dose distribution of about 1% in both phantom. As a result of using the F1 filter, the difference in dose distribution between the PLA phantom and the RW3 phantom was 2.71 ± 1.99%, and as a result of using the F2 filter, there was a difference of 2.6 ± 0.52%. These results show that the selection of the filtration filter can be different depending on the material.

## Conclusion

We tried to develop an optimized experimental set-up for TLI in rat lung transplantation. As a part of the effort, we made a sophisticated rat phantom and fabricated a shielding apparatus that can be controlled according to rat size. We verified that head, limbs, and tail were effectively shielded. We also tried to achieve accurate dose delivery even when performing TLI using a real rat with an image-guided system.

## Data Availability Statement

The original contributions presented in the study are included in the article/Supplementary Material, further inquiries can be directed to the corresponding authors.

## Author Contributions

All authors certify that they have participated sufficiently in the work to take public responsibility for the content, including participation in the concept, design, analysis, writing, or revision of the manuscript. Furthermore, each author certifies that this material or similar material has not been submitted to or published in any other journal.

## Conflict of Interest

The authors declare that the research was conducted in the absence of any commercial or financial relationships that could be construed as a potential conflict of interest.

## Publisher's Note

All claims expressed in this article are solely those of the authors and do not necessarily represent those of their affiliated organizations, or those of the publisher, the editors and the reviewers. Any product that may be evaluated in this article, or claim that may be made by its manufacturer, is not guaranteed or endorsed by the publisher.

## References

[1] Krupnick AS Lin X Li W Okazaki M Lai J Sugimoto S. Orthotopic mouse lung transplantation as experimental methodology to study transplant and tumor biology. Nat Protoc. (2013) 4:1–18. 10.1038/nprot.2008.218 19131960 PMC3848695

[2] Diamond DA Michalski JM Lynch JP Trulock EP. Efficacy of total lymphoid irradiation for chronic allograft rejection following bilateral lung transplantation. Int J Radiat Oncol Biol Phys. (1998) 41:795–800. 10.1016/S0360-3016(98)00113-8 9652840

[3] Najarian JS Ferguson RM Sutherland DE Slavin S Kim T Kersey J. Fractionated total lymphoid irradiation as preparative immunosuppression in high risk renal transplantation. Clinical and immunological studies. Ann Surg. (1982) 196:442–52. 10.1097/00000658-198210000-00007 6812511 PMC1352705

[4] Mckay C Knight KA Wright C. Beyond cancer treatment – A review of total lymphoid irradiation for heart and lung transplant recipients. J Med Radiat Sci. (2014) 61:202–9. 10.1002/jmrs.63 26229656 PMC4175854

[5] Tallaj JA Pamboukian SV George JF Brown RN Pajaro OE Bourge RC. Total lymphoid irradiation in heart transplantation: Long-term efficacy and survival-an 18-year experience. Transplantation. (2011) 92:1159–64. 10.1097/TP.0b013e318231e9d3 22015463

[6] Renzo C Michele Z Stefano V Stefano A Salvina B Gianni T. Helical tomotherapy targeting total bone marrow after total body irradiation for patients with Q20 relapsed acute leukemia undergoing an allogeneic stem cell transplant. Radiother Oncol. (2011) 98:382–6. 10.1016/j.radonc.2011.01.016 21339008

[7] Xie T Zaidi H. Development of computational small animal models and their applications in preclinical imaging and therapy research. Med Phys. (2015) 43:111–31. 10.1118/1.4937598 26745904

[8] Bax JS Waring CSR Sherebrin S Stapleton S Hudson TJ Jaffray DA. 3D image-guided robotic needle positioning system for small animal interventions. Med Phys. (2013) 40:11909. 10.1118/1.4771958 23298100

[9] Kim H Fabien J Zheng Y. Establishing a process of irradiating small animal brain using a CyberKnife and a microCT scanner. Med Phys. (2014) 41:021715. 10.1118/1.4861713 24506606

[10] Hoogenhout J. Total lymphoid irradiation in the wistar rat: technique and dosimetry. Int J Radiat Oncol Biol Phys. (1983) 113–7. 10.1016/0360-3016(83)90220-1 6841171

[11] Halperin EC Knechtle S Abernethy K. The influence of dose and dose rate of total lymphoid irradiation in the rat cardiac allograft model. Radiother Oncol. (1987) 9:311–8. 10.1016/S0167-8140(87)80152-4 3317525

[12] Prise KM Verhaegen F. Small animal image-guided radiotherapy. Br J Radiol. (2017) 90.1069: 20160905. 10.1259/bjr.20160905 27998187 PMC5605045

[13] Wong J Armour E Kazanzides P Iordachita I Tryggestad E Deng H. High-resolution, small animal radiation research platform with x-ray tomographic guidance capabilities. Int J Radiat Oncol Biol Phys. (2008) 71:1591–9. 10.1016/j.ijrobp.2008.04.025 18640502 PMC2605655

[14] Clarkson R Lindsay P Ansell S Wilson G Jelveh S Hill R. Characterization of image quality and image-guidance performance of a preclinical microirradiator. Med Phys. (2011) 38:845–56. 10.1118/1.3533947 21452722 PMC3188651

[15] Verhaegen F Dubois L Gianolini S Hill MA Karger CP Lauber K. ESTRO ACROP: technology for precision small animal radiotherapy research: Optimal use and challenges. Radiother Oncol. (2018) 126:471–8. 10.1016/j.radonc.2017.11.016 29269093

[16] Mark A Hill Borivoj Vojnovic. Implications of respiratory motion for small animal image-guided radiotherapy. BJR. (2017) 90:20160482 10.1259/bjr.20160482 27384471 PMC5605024

[17] van der Heyden B van Hoof SJ Schyns LEJR. The influence of respiratory motion on dose delivery in a mouse lung tumor irradiation using the 4D MOBY phantom. BJR. (2017) 90:20160419. 10.1259/bjr.20160419 27626324 PMC5605015

[18] Hill RP. The changing paradigm of tumour response to irradiation. Br J Radiol. (2017) 90:20160474. 10.1259/bjr.20160474 27416998 PMC5605022

[19] Corroyer-Dulmont A Falzone N Kersemans V Thompson J Hill M Allen PD. MRI-guided radiotherapy of the SK-N-SH neuroblastoma xenograft model using a small animal radiation research platform. Br J Radiol. (2017) 90:20160427. 10.1259/bjr.20160427 27524406 PMC5605018

[20] Ma C-M Coffey CW DeWerd LA. AAPM protocol for 40-300 kV x-ray beam dosimetry in radiotherapy and radiobiology. Med Phys. (2001) 28:868–93. 10.1118/1.1374247 11439485

[21] Andreo P Burns D Hohlfeld K Huq MS Kanai T Laitano F. IAEA TRS-398-Absorbed dose determination in external beam radiotherapy: an international code of practice for dosimetry based on standards of absorbed dose to water. International Atomic Energy Agency. (2000) 18:35–36.

[22] Pavoni JF Neves-Junior WFP Da Silveira MA Haddad CMK Baffa O. Evaluation of a composite Gel-Alanine phantom on an end-to-end test to treat multiple brain metastases by a single isocenter VMAT technique. Med Phys. (2017) 44:4869–79. 10.1002/mp.12400 28594461

[23] Penev KI Mequanint K. Multifactorial study and kinetics of signal development in ferrous-methylthymol blue-gelatin gel dosimeters. Med Phys. (2017) 44:1948–57. 10.1002/mp.12201 28273352

[24] Pisaturo O Miéville F Tercier P-A Allal AS. TransitQA – a new method for transit dosimetry of Tomotherapy patients. Med Phys. (2017) 45:438–47. 10.1002/mp.12672 29136280

[25] Deshpande S Blake SJ Xing A Metcalfe PE Holloway LC Vial P. A simple model for transit dosimetry based on a water equivalent EPID. Med Phys. (2018) 45:1266–75. 10.1002/mp.12742 29314080

[26] Azimi R Alaei P Spezi E Hui SK. Characterization of an orthovoltage biological irradiator used for radiobiological research. J Radiat Res. (2015) 56:485–92. 10.1093/jrr/rru129 25694476 PMC4426923

